# A Biomechanical Comparison of Two Intramedullary Implants for Subtrochanteric Fracture in Two Healing Stages: A Finite Element Analysis

**DOI:** 10.1155/2015/475261

**Published:** 2015-02-23

**Authors:** Xinlei Wu, Ming Yang, Lijun Wu, Wenxin Niu

**Affiliations:** ^1^Institute of Digitized Medicine, Wenzhou Medical University, Wenzhou, Zhejiang 325035, China; ^2^Department of Orthopaedics & Traumatology, Peking University People's Hospital, Beijing 100044, China; ^3^Laboratory of Sports Biomechanics, Yang Zhi Rehabilitation Hospital, Tongji University School of Medicine, Shanghai 2001619, China

## Abstract

*Background*. The biomechanical effect of two implants, namely, proximal femoral nail antirotation for Asia (PFNA-II) and Expert Asian Femoral Nail (A2FN), for treating subtrochanteric fracture during healing stages, is still unclear. *Methods*. A 3D finite element model of an intact femur was constructed and validated. The fractured and postoperative models were accordingly produced. The postoperative models were loaded with the peak joint forces during gait for the soft and hard callus stages. The effects of stress distribution on the implants, femoral head and callus, and the deformation of the proximal femur were examined. *Results*. Both implants showed similar biomechanical effect in two healing stages. As the healing duration increased, the von Mises stress of two implants and the tensile stress of the femoral head decreased, whereas the compressive stress of the femoral head increased. However, the PFNA-II operation resulted in higher stress on the implant, lower stress on the proximal femur, and lower compressive stress and higher tensile stress on the callus than A2FN operation. *Conclusions*. The A2FN implant may provide a biomechanically superior construct for subtrochanteric fracture healing. However, the upper screw of the A2FN implant may be more likely to be loose in the healing process.

## 1. Introduction

Subtrochanteric fracture (STF) normally occurs at or below the lesser trochanter in the proximal femur [1]. This always leads to many complications, high mortality rate, and a decreased quality of life. It has been reported that elderly Asians have a significantly higher STF incidence than white people (10% versus 1.4%) [2]. Along with the aging of the population in many Asian nations, the number of STF patients will be rapidly increasing in the next decades.

STF poses a great challenge to the fixation approaches, because the subtrochanteric region is an area of high stress concentration [3]. This region is also subject to multiple muscle forces, which make anatomic reduction of the fracture difficult. Intramedullary device fixation has now become the preferred method of treatment for the majority of unstable femoral fractures, since it is biomechanically superior to extramedullary implants in lowering the moment arm on the implant and bearing more weight during daily activities [4, 5].

A proximal femoral nail antirotation for Asia (PFNA-II) has been developed especially for Asian patients. Some clinical studies have previously reported that the short-term clinical outcomes of PFNA-II are satisfactory in most patients, and it provides an anatomy matched with the narrower and shorter femurs of Asians and contributes to decreased complications [6]. Another intramedullary device is the Expert Asian Femoral Nail (A2FN), which has been already used in Asian elderly patients, and the clinical outcomes also have been relatively satisfactory.

In the last years, numerous finite element (FE) models of femur implant composite have been created to analyze the biomechanical effect of different fixations [7–10]. Most of these studies only paid attention to the immediate postoperative biomechanical effect of different implants. Actually, the biomechanical problem is even important in the healing process, which includes the stages of inflammation, soft callus, hard callus, and remodelling [11]. Though it is known that many surgeries fail in the healing process, the biomechanical effect of the different implants in the process is still unclear.

Therefore, the aim of this study was to compare the biomechanical effects of two different intramedullary fixations for the treatment of STF. A subject-specific finite element model of the femur was constructed for an elderly Asian, and two different intramedullary fixations were simulated to investigate the biomechanical effects in the soft callus and hard callus stages.

## 2. Methods

### 2.1. Finite Element Modelling

A woman (age: 65 years; body mass: 70 kg; height: 158 cm) was CT-scanned at 1.0 mm transverse resolution in 1.0 mm increments. The geometry of the femur and medullary canal surfaces were created by extracting the sequential cross section of bony femur in mimics (version 10.0, Materialise Leuven, Belgium). The fractured model (AO classification 32-A3.1) was then produced at 3-cm below the level of the lesser trochanter. The modelling process was detailed elsewhere [12–14].

The model was meshed with 4-node tetrahedron elements. Convergence tests were performed to decide on the optimum maximum element size. The callus was simulated in two different healing stages through different material properties. The elastic moduli of the callus were, respectively, assigned as 5,000 MPa in the soft callus stage (SCS) and 15,000 MPa in the hard callus stage (HCS) [11]. A recent study showed that the FE computation was greatly affected by the assignation of the elastic modulus [12]. Therefore, the material properties of the elderly intact femur were accurately assigned on an element-by-element basis using the density-gray and modulus-density relationship as follows [15]:

(1) $$\begin{array}{c} \rho =-13.4+1017\times \text{Gv},\\ E=-388.8+5925\times \rho , \end{array}$$

where Gv is gray value; *ρ* is the element density; and *E* is the elastic modulus. The geometry of PFNA-II and A2FN implant configurations were constructed in CATIA (version 5R19, Dassault Systems, France). Then they were imported into the intact FE model and were assembled to the proper locations in HyperMesh (version 10.0, Altair Hyperworks, USA). Two implants were both made of titanium alloy and defined as linearly elastic (elastic modulus *E* = 110 GPa; and Poisson's ratio *v* = 0.3).

### 2.2. Contact Surfaces

**Figure 1:**
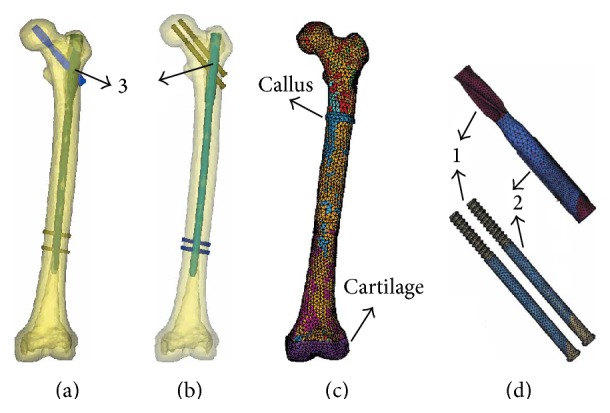
The finite element models (a, b) PFNA-II and A2FN implants fixing the femur models; (c) FE model of the elderly femur with the element-by-element material properties; (d) the femoral neck load carrier (PFNA-II blade/A2FN screws). Three different contact conditions in the bone-implant-construct (1 = contact elements, 2 = sharing common nodes, and 3 = coupling nodes' degree of freedom).

As shown in Figure 1, the contact relationships were processed with three different approaches: (1) contact elements; (2) sharing common nodes; and (3) coupling nodes' degree of freedom. Because a relative tangential motion may occur in certain regions between the bone and the middle part of the femoral neck load carrier (PFNA-II's blade/A2FN's screws), these interfaces were simulated by contact pairs (CONTA173 and TARGE170) with a friction factor of 0.3 [16]. The interfaces between the head of the femoral neck load carrier and femoral head were simplified using tetrahedron elements, whose nodes were shared with each other. The interfaces of the main intramedullary nail and the femoral neck load carrier were coupled for the nodes on the internal surface of the main nail holes. The numbers of element and node for both models were listed in Table 1.

### 2.3. Model Validation

To validate the FE model, we reconstructed an intact femur FE model and made an analysis to compare with the published experimental data [17]. According to the* in vitro *experiment by Papini et al. [17], the distal end of the femur model was fully constrained on the cartilage. A vertical load of 1.5 kN was applied on the femoral head. The axial stiffness of our FE computation was 0.94 kN/mm and was in the measurement interval (0.76 ± 0.26 kN/mm) [17]. Considering the individual differences, the FE model was satisfactorily validated.

### 2.4. Boundary and Loading Conditions

The Cartesian coordinate system was generated from the CT scanning and replotted in ANSYS software (version 11.0, ANSYS, USA). The *XY* plane was settled parallel to transverse plane, and the *X*-axis was formed by the intersection of the coronal and the transverse plane. The distal end of the femur model was fully constrained on the cartilage. The peak joint force in the adult gait cycle was considered. As a reference situation presented by Taylor et al. [18], the hip joint reaction force and the muscle forces of the abductor, iliopsoas, and vastas were 2,872 N, 1,237 N, 771 N, and 1,200 N, respectively.

## 3. Results

### 3.1. Stress Distribution on Implants

**Figure 2:**
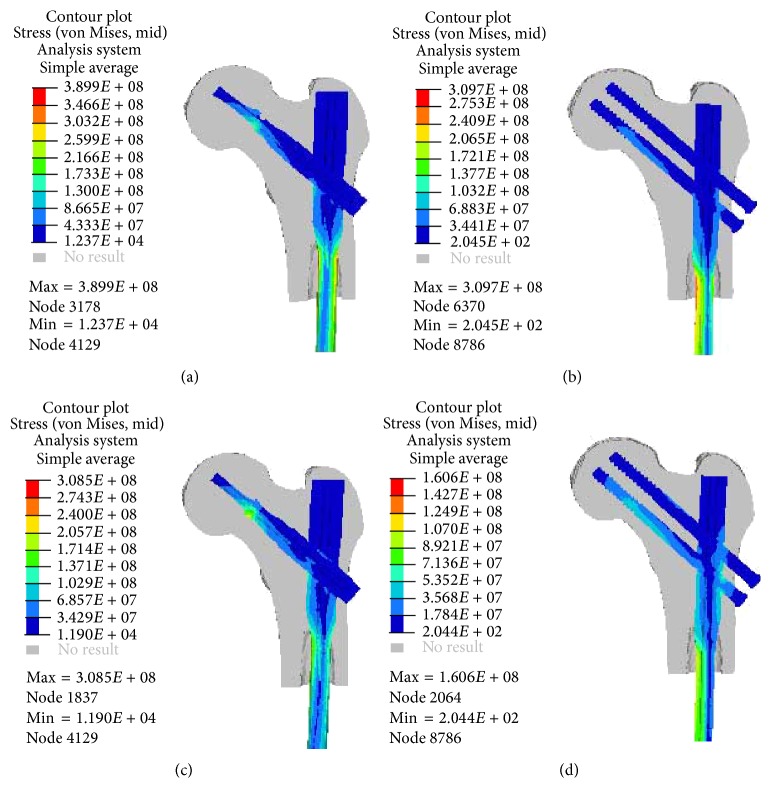
Comparison of von Mises stresses of the PFNA-II and A2FN implants in the two different healing stages from section view (a, b) in the soft callus stage, (c, d) in the hard callus stage.

The von Mises stresses induced on the PFNA-II and A2FN implants in two healing stages were shown in Figure 2. The load transfer mechanisms were similar for two implants in the same healing stage. In SCS, the stress was concentrated at both medial and lateral sides of the main intramedullary nail's neck. In HCS, the stress was concentrated at the hole between the intramedullary nail and the femoral neck load carrier (Figures 2(c) and 2(d)). The stress concentrations were also on the edges of the blade in PFNA-II model and the screw thread areas in A2FN model. The peak von Mises stress of the two implants' femoral neck load carrier did not change obviously between two healing stages, but that of the two implants' main intramedullary nails both decreased as the healing duration increased. As listed in Table 2, the PFNA-II implant experienced higher stress than the A2FN from a global aspect. The peak von Mises stress of the A2FN decreased by 54.1% from SCS to HCS. The decreasing ratio was obviously more than that of the PFNA-II (20.9%). Particularly, the stress distributions on the A2FN's screws were nonuniform. The stress was higher on the lower screw than on the upper one (Figures 2(a) and 2(b)).

### 3.2. Stress Distribution on Proximal Femurs

As listed in Table 3, the peak principal stresses in two healing stages were compared between two implants. The peak tensile stress and compressive stress of the PFNA-II model were both more than those of the A2FN model in both healing stages. However, the peak von Mises stress on the femur was slightly higher in the A2FN model than the PFNA-II model.

### 3.3. Displacement Pattern

**Figure 3:**
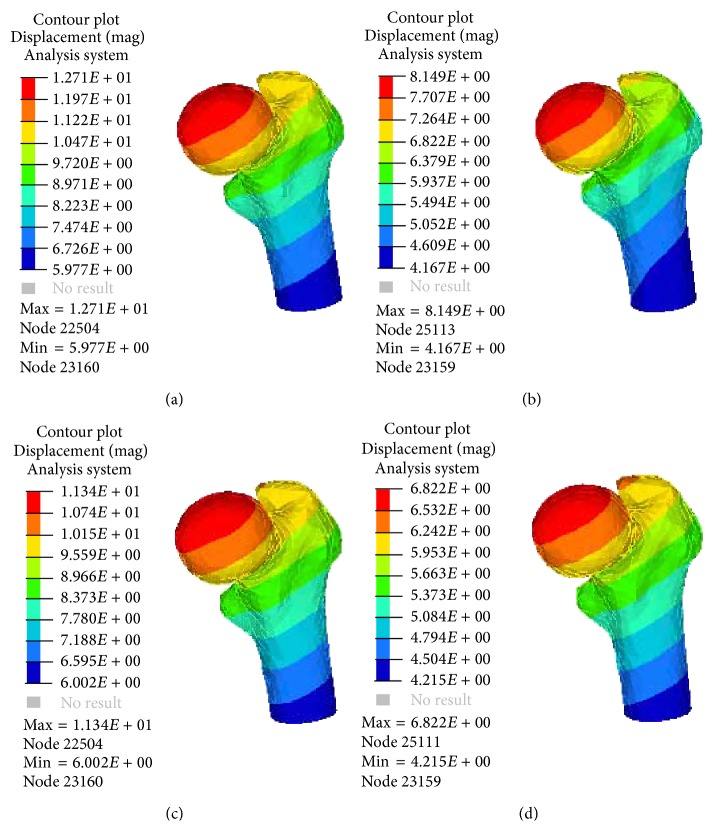
Deformation of the treated femoral head in the two different healing stages. (a) PFNA-II model in SCS; (b) A2FN model in SCS; (c) PFNA-II model in HCS; (d) A2FN model in HCS.

A comparison of the peak displacements of the femur head was shown as in Figure 3. The model displacements in SCS were more than those in HCS. The displacements of the PFNA-II model were more than the A2FN model in the same healing stage. The value of the medial-lateral displacement was the smallest and the values in both healing stages were similar, while the anterior-posterior and vertical displacements changed obviously. As shown in Table 4, the anterior-posterior displacement of the PFNA-II decreased by 10.8% from SCS to HCS, but that of the A2FN decreased by 23.7%.

### 3.4. Stress State of the Callus

**Figure 4:**
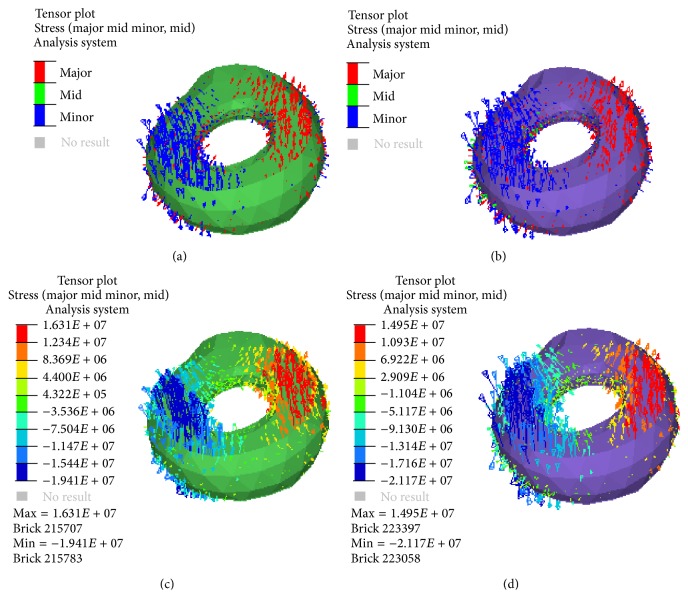
Tensor plot of the principal stresses of the two models' callus in the two different healing stages (a, b) with direction; the red colour represents compressive stress and the blue colour represents tensile stress; (c, d) with value. (a) PFNA-II model in SCS; (b) A2FN model in SCS; (c) PFNA-II model in HCS; (d) A2FN model in HCS.

The tensile and compressive stresses of the callus in four models were compared and plotted as Figure 4. The stress states of the callus were also similar for two models. In both stages, the compressive stress was obvious on the medial callus, while the tensile stress is mainly on the lateral side. In HCS, the compressive stress of the callus in the A2FN model was more than that in the PFNA-II model, but the tensile stress of the callus in the A2FN model was less than that in the PFNA-II model.

## 4. Discussion

Intramedullary fixation is the primary surgical treatment for unstable STF. PFNA-II and A2FN were both developed especially for Asian patients. Their curative effects of the implants were relatively satisfied in most patients. They were similar in the shape of their main intramedullary nails, which were both smaller radius and larger bow curvature. It could be matched with the narrower and shorter femurs of Asians. However, the major difference between two implants is that the PFNA-II has a helical neck blade with large surface area providing rotational and angular stability [19, 20], whereas the A2FN has two cephalocervical screws in an integrated mechanism to support compressive and rotational loading from the femoral head.

FE analysis showed that PFNA-II and A2FN devices had some similar biomechanical effect for the treatments of STF between SCS and HCS. The two intramedullary fixations, to some extent, both share the axial load and transfer to the distal femur. As the healing duration increased, the rigidity of the callus changed. Compared with the soft callus, the hard callus shared more external loads and caused fewer loads on the implants. Also, the compressive stress on the medial side of the femoral head increased, whereas on the lateral side the tensile stress it decreased. Meanwhile, the displacements of these implant-systems decreased and the stabilities were improved. Particularly, the stress concentration of the intramefullary nail transferred from its neck to the hole between the nail and these femoral neck load carriers. When the axial force of the implant decreased, the tangential force of the hole between the intramefullary nail and these femoral neck load carriers also decreased. Therefore, the femoral neck load carrier would become more loosening as the fracture healing duration increased. Moreover, the walking would lead to increasing of stress on the callus and implants in the SCS, compared to the HCS. This would potentially delay the healing duration and increase the risk of reduction malposition and even implant failure. Therefore, the walking should be avoided in the early healing stage.

However, there were several biomechanical differences between the PFNA-II and A2FN models. The strength of the implants is one of the factors for the success of the surgery. The peak von Mises stress of all the components of the A2FN was much less than those of the PFNA-II in the same healing stage. It is mainly due to structural differences between the one blade and the two screws. The A2FN, consisting of two screws and an intramedullary nail, could share the external loads more effectively. This structure resulted in lower stress on the main intramedullary nail. The distance between the two A2FN's screws was approximately 20.0 mm. It was longer than the diameter of the PFNA-II's blade (11.6 mm). Thus, the A2FN could play its role more effectively with the larger support space. On the other hand, the PFNA-II had to support the same load with a single blade. It would result in higher stress in the main intramedullary nail. Undoubtedly, the higher stress is dangerous for the implant.

The overall stability of the A2FN implant was superior to the PFNA-II fixation. As mentioned above, the screws may be loose mainly in the medial-lateral direction. Weil et al. [21] reported that the medial migration of the femur is a common complication in the intramedullary fixation for STF. Thus, the stability of the bone-implanted system should be further considered in the medial-lateral direction.

Excessive compressive stress at the medial site and tensile stress at the lateral site will cause coxa vara. These stresses all had no significant difference between two implants in our computation. However, the compressive stress of the callus in the A2FN model was more than that in the PFNA-II model, but the value of the tensile stress was smaller in the A2FN model. The compressive stress in a certain interval at the fracture site would promote fracture healing, but excessive tensile stress could enlarge the fracture gap and even caused nonunion [22].

Furthermore, the stress of the upper screw in A2FN model was less than that of its lower screw. It could avoid the risk of these screws cut-out of the femoral head. However, in the HCS, the pressure of the main intramedullary nail in the A2FN significantly reduced as the hard callus supported more loads. It resulted in less friction between the screws and main intramedullary nail. Thus, the upper screw could become more likely to loosen and further increase the stress of the lower screw.

There are also some limitations in this study. Firstly, the materials of cancellous and cortical bones were simplified. The relationship between CT grays and elastic modulus in this paper was reported elsewhere [15]. When it was used to simulate the material properties of elderly femur, there may be some deviation. Secondly, it is difficult to determine what exactly happens at the interface between the bone and implants after surgery and the change of the interface in the healing process. Thirdly, the callus volume was assumed in this study. The callus formation changes in volume in different healing stage. However, it does not affect the result, because the two models were under the same simplified conditions.

## 5. Conclusions

Compared with the A2FN, the PFNA-II implant experienced higher stress but resulted in less stress on the proximal femur. The femur implanted by A2FN has more stability than PFNA-II. Considering the stress state of the callus, the A2FN may be more suitable for the healing than PFNA-II. As a suggestion, the screws of A2FN may be more likely to be loose as the healing duration increases.

## Figures and Tables

**Table 1:** Numbers of elements, nodes, and contact elements in the PFNA-II and A2FN models.

Models	PFNA-II		A2FN	
	Element number	Node number	Element number	Node number
Femur	51666	13157	133789	27192
Implant configurations				
Main intramedullary nail	32149	8628	46280	11011
Screws/blade	15654	4195	14658	4396
Distal locking screws	5579	1472	6353	1635
Contact elements	6370	3437	6099	2967

**Table 2:** The peak von Mises stress of the implant configurations in two healing stages (MPa).

Healing stage	Implant	Blade/hip screw	Main intramedullary nail
Soft callus	PFNA-II	243.7	389.9
	A2FN	66.9	309.7

Hard callus	PFNA-II	243.6	308.5
	A2FN	64.0	142.0

**Table 3:** Comparison of peak stresses of the femoral head between two implants (MPa).

Healing stage	Implant	Principal stress		von Mises stress
		Tensile	Compressive	
Soft callus	PFNA-II	40.4	−42.2	45.2
	A2FN	30.5	−33.1	46.3

Hard callus	PFNA-II	39.2	−45.2	46.3
	A2FN	28.2	−39.1	47.3

**Table 4:** The maximum displacements of the proximal femur in two healing stages (mm).

Healing stage	Implant	UM	UX	UY	UZ
Soft callus	PFNA-II	12.7	3.9	12.0	−5.5
	A2FN	8.1	3.7	7.6	−3.7

Hard callus	PFNA-II	11.3	3.8	10.7	−4.2
	A2FN	6.8	3.6	5.8	−2.2

## References

[1] Li F., Sang W., Wang Q., Huang J., Lu H. (2011). Subtrochanteric fracture treatment: a retrospective study of 46 patients. *Medical Principles and Practice*.

[2] Calder S. J., Anderson G. H., Harper W. M., Gregg P. J. (1994). Ethnic variation in epidemiology and rehabilitation of hip fracture. *British Medical Journal*.

[3] Ekström W., Németh G., Samnegård E., Dalen N., Tidermark J. (2009). Quality of life after a subtrochanteric fracture. A prospective cohort study on 87 elderly patients. *Injury*.

[4] Barquet A., Mayora G., Fregeiro J., López L., Rienzi D., Francescoli L. (2004). The treatment of subtrochanteric nonunions with the long Gamma nail: twenty-six patients with a minimum 2-yeaar follow-up. *Journal of Orthopaedic Trauma*.

[5] Saarenpää I., Heikkinen T., Jalovaara P. (2007). Treatment of subtrochanteric fractures. A comparison of the Gamma nail and the dynamic hip screw: short-term outcome in 58 patients. *International Orthopaedics*.

[6] Lv C. L., Fang Y., Liu L. (2011). The new proximal femoral nail antirotation-Asia: early results. *Orthopedics*.

[7] Helwig P., Faust G., Hindenlang U. (2009). Finite element analysis of four different implants inserted in different positions to stabilize an idealized trochanteric femoral fracture. *Injury*.

[8] Wang L. Z., Zhao F., Han J. Y., Wang C., Fan Y. B. (2012). Biomechanical study on proximal femoral nail antirotation (PFNA) for intertrochanteric fracture. *Journal of Mechanics in Medicine and Biology*.

[9] Gong H., Wang L., Zheng D., Fan Y. (2012). The potential application of functionally graded material for proximal femoral nail antirotation device. *Medical Hypotheses*.

[10] Sahli A., Benbarek S., Wayne S., Bouiadjra B. A. B., Serier B. (2014). 3D crack behavior in the orthopedic cement mantle of a total hip replacement. *Applied Bionics and Biomechanics*.

[11] Shefelbine S. J., Simon U., Claes L. (2005). Prediction of fracture callus mechanical properties using micro-CT images and voxel-based finite element analysis. *Bone*.

[12] Niu W. X., Wang L. J., Feng T. N., Jiang C. H., Fan Y. B., Fan Y. B. (2013). Effects of bone Young's modulus on finite element analysis in the lateral ankle biomechanics. *Applied Bionics and Biomechanics*.

[13] Ni M., Weng X. H., Mei J., Niu W. X. Primary stability of absorbable screw fixation for intra-articular calcaneal fractures.

[14] Niu W. X., Zhang T. T., Jiang M., Jiang C. H., Fan Y. B. (2014). An in-vitro and finite element study of load redistribution in the midfoot. *Science China Life Sciences*.

[15] Mann K. A., Lee J., Arrington S. A., Damron T. A., Allen M. J. (2008). Predicting distal femur bone strength in a murine model of tumor osteolysis. *Clinical Orthopaedics and Related Research*.

[16] Nuño N., Amabili M., Groppetti R., Rossi A. (2002). Static coefficient of friction between Ti-6A1-4V and PMMA for cemented hip and knee implants. *Journal of Biomedical Materials Research*.

[17] Papini M., Zdero R., Schemitsch E. H., Zalzal P. (2007). The biomechanics of human femurs in axial and torsional loading: comparison of finite element analysis, human cadaveric femurs, and synthetic femurs. *Journal of Biomechanical Engineering*.

[18] Taylor M. E., Tanner K. E., Freeman M. A. R., Yettram A. L. (1996). Stress and strain distribution within the intact femur: compression or bending?. *Medical Engineering and Physics*.

[19] Strauss E., Frank J., Lee J., Kummer F. J., Tejwani N. (2006). Helical blade versus sliding hip screw for treatment of unstable intertrochanteric hip fractures: a biomechanical evaluation. *Injury*.

[20] Huang Y. F., Zhang C. L., Luo Y. (2013). A comparative biomechanical study of proximal femoral nail (InterTAN) and proximal femoral nail antirotation for intertrochanteric fractures. *International Orthopaedics*.

[21] Weil Y. A., Gardner M. J., Mikhail G., Pierson G., Helfet D. L., Lorich D. G. (2008). Medial migration of intramedullary hip fixation devices: a biomechanical analysis. *Archives of Orthopaedic and Trauma Surgery*.

[22] de Vries J. S., Kloen P., Borens O., Marti R. K., Helfet D. L. (2006). Treatment of subtrochanteric nonunions. *Injury*.

